# Changes of von Willebrand Factor during Pregnancy in Women with and without von Willebrand Disease

**DOI:** 10.4084/MJHID.2013.052

**Published:** 2013-07-16

**Authors:** Giancarlo Castaman

**Affiliations:** Hemophilia and Thrombosis Center, Department of Cell Therapy and Hematology, San Bortolo Hospital, Vicenza, Italy.

## Abstract

Delivery in von Willebrand disease (VWD) represents a significant hemostatic challenge because of the variable pattern of changes observed during pregnancy of von Willebrand factor (VWF) and factor VIII (FVIII), the protein carried by VWF. Since a wide heterogeneity of phenotypes and of the underlying pathophysiological mechanisms is associated with this disorder, a prompt and careful evaluation of pregnant women with VWD is requested in order to plan the most appropriate treatment at time of parturition. VWF and FVIII increase significantly during pregnancy in normal women, already within the first trimester, reaching levels by far >100 U/dL by the time of parturition. Women with VWD, levels at baseline of VWF and FVIII >30 U/dL have us a high likelihood to achieve normal levels at the end of pregnancy; thus specific anti-hemorrhagic prophylaxis is seldom required. Women with basal level <20 U/dL usually have a poor increase since most of these women carry mutations associated with increased VWF clearance or are compound heterozygous for different VWF mutations; that prevent the achievement of satisfactory hemostatic levels. While women with mutations associated with increased clearance show a full, albeit transitory correction of their hemostatic deficiency after desmopressin administration, compound heterozygous need replacement therapy because they do not respond well to this agent. Patients with abnormal VWF:RCo/VWF:Ag ratio at baseline (e.g. <0.6), typically associated with type 2 VWD, maintain the abnormality throughout pregnancy and VWF:RCo usually does not attain safe levels ≥50 U/dL. These women require replacement therapy with VWF-FVIII concentrates. Delayed post-partum bleeding may occur when replacement therapy is not continued for some days. Tranexamic acid may be useful at discharge to avoid excessive lochia.

## Introduction

Von Willebrand disease (VWD) is a common autosomal inherited bleeding disorder caused by quantitative or qualitative defects of von Willebrand factor (VWF), a multi-adhesive protein which binds platelets to exposed subendothelium and carries factor VIII (FVIII) in circulation.^1^,^2^ As a consequence, FVIII, the protein deficient in hemophilia A, may be variably reduced. Clinical manifestations are mainly represented by mucous membrane and soft tissue bleeding and their severity is variable depending on the degree of VWF and FVIII reduction.^2^ Women with VWD are at particularly high risk of bleeding because of menorrhagia and delivery.

Women may undergo to naturally-occurring physiological events (menstruation, pregnancy and parturition) that may cause undue bleeding complications even in absence of a specific bleeding disorder. At the time of delivery, several obstetric complications may cause bleeding with or without associated hematologic alterations.^3^ A standard definition of post-partum hemorrhage (PPH) considers the threshold of 500 mL blood loss for vaginal delivery and of 1,000 mL for caesarean section.^4^ Rates of PPH in Western Countries based on hospital discharge may range from 3 to 6%, while cases needing blood transfusion are as low as 1%.^3^

Uterine atony is still the leading cause of hemorrhage at parturition accounting for approximately 75% of all cases with PPH and may be burdened with a high risk of being transfused or even death in otherwise healthy women.^3^,^5^ Of course, these complications may add to the inherent risk of bleeding in women with inherited bleeding disorder.

## Risk of bleeding at parturition in women with von Willebrand disease

There are conflicting results in the literature about the correct estimation of the risk and the severity of bleeding at parturition in women with VWD. Historical series reported percentages of VWD women with post-partum bleeding ranging from 15 to 60%.^6^–^10^ This uncertainty is also reported when women with wide range of basal FVIII and VWF are included among the type 1 patients. In the MCMDM-1VWD study no difference of bleeding risk at parturition was observed between women with VWD and their normal relatives.^11^ In a recent large case-control study from the USA including 4067 deliveries in women with VWD based on all pregnancy-related discharge codes for the years 2000–2003, they were more likely to experience PPH (OR 1.5; 95% CI 1.1–2.0) and had a fivefold increased risk of being transfused (OR 4.7; 95% CI 3.2–7.0) compared with women without VWD.^12^ However, no data about anti-hemorrhagic prophylaxis and adequacy of treatment was available and thus it is not possible to give a reliable figure of the risk. Furthermore, in this study, women with VWD were 10 times more likely to experience other ante-partum bleeding (OR 10.2; 95% CI 7.1–14.6),^12^ but this latter data was not confirmed in a recent study where the risk of PPH in women with VWD was similar to that of normal women.^13^

Only a single study has tackled the estimation of the risk of bleeding occurring in women before the diagnosis of VWD was made.^14^ This is important since in this case no prophylactic anti-hemorrhagic treatment was administered and thus the results reflect the unmodified risk of bleeding in these women. In the IMS study, for the first time a structured bleeding questionnaire was used and grading the severity of the symptoms allowed the estimation of the risk of bleeding and its severity between women belonging to families with a clear autosomal dominant type 1 VWD and normal women (Table 1).^14^ The risk of receiving blood transfusion, undergoing uterine dilation/curettage or suturing was about 8-fold greater in VWD women. Furthermore, the risk of hysterectomy for post-partum bleeding was extremely high. Thus, women with VWD without treatment have a clear risk of post-partum bleeding if untreated. However, again this risk may vary depending on the pathophysiologic mechanisms causing VWF deficiency and the inherent likelihood to achieve normal levels at the end of pregnancy (see below).

## Factor VIII and von Willebrand factor changes during pregnancy in normal women and women with von Willebrand disease

Pregnancy is considered as a hypercoagulable state because the increase of several hemostatic factors is observed. Factor VII, X, fibrinogen and plasminogen activator inhibitor type 1 do increase, but free protein S decrease.^15^ The common view considers these changes to be in preparation for the hemostatic challenge of delivery. Also VWF and FVIII increase significantly during pregnancy in normal women and the greatest increase is evident during the third trimester, with levels far exceedingly >100 U/dL by the time of parturition.

A progressive increase of these moieties is also evident in women with type 1 VWD, the partial quantitative deficiency of the disorder, with levels >50 U/dL in the third trimester.^16^ However, owing to the wide heterogeneity of phenotypes and genotypes underlying also this type, this general statement needs to be interpreted cautiously and careful evaluation of any pregnant woman with a diagnosis of VWD is recommended. In general, women with levels at baseline of VWF and FVIII >30 U/dL show usually a high likelihood to achieve normal levels at the end of pregnancy.^17^ Women with basal level <20 U/dL usually have a poor increase since most of these women carry mutations associated with increased VWF clearance or are compound heterozygous for different VWF mutations which prevent the achievement of physiologically satisfactory hemostatic levels.^18^,^19^ Some frequent autosomal dominant mutations (e.g. R1205H, C1130F) are associated with an increased clearance of VWF, as documented by an increased VWF propeptide/VWF:Ag ratio,^20^ which do not allow the achievement of significant levels at the end of pregnancy.^21^,^22^ Similarly, women with compound heterozygosity for null and missense mutations, associated with clearly measurable FVIII/VWF levels do not achieve significant improvements during pregnancy.^19^ Since the genetic background which is highly predictive of the type of response to desmopressin and changes during pregnancy is no available for most of these patients,^19^ a careful monitoring during pregnancy or at least during the third trimester is highly recommended to identify those who will need specific treatment.

Type 2A VWD is characterized by the lack of high molecular weight multimers and an abnormal VWF:RCo/VWF:Ag (<0.6).^19^ During pregnancy, multimer abnormality does not correct. A significant increase of FVIII and VWF:Ag may occur, but VWF:RCo remains markedly reduced.

Worsening of an already existing thrombocytopenia is the most important change observed in type 2B VWD women during pregnancy because an increased release of abnormal multimers with enhanced affinity for glycoprotein Ib on platelet surface occurs.^23^,^24^ However, the severity of this phenomenon is strongly dependent on the type of mutations in A1 domain of VWF responsible for this type, with some mutations showing normal platelet count (e.g., P1266L) while others being associated with severe thrombocytopenia (e.g. R1308C, M1316V).^25^ In any case, platelet count should be also closely monitored during pregnancy in women with this type.

Type 2M women usually show a significant correction of FVIII and VWF:Ag, while VWF:RCo does not attain levels around 50 U/dL, as is usually observed after desmopressin.^19^,^26^

In type 2N, normalization of FVIII, which is more reduced compared to VWF in this type, usually occurs in women heterozygous or homozygous for the most frequent mutation R854Q responsible for this type,^27^ while information with the rarer mutations is still scanty.

Women with typical type 3 VWD do not show any change during VWF because their endothelial VWF stores are lacking.

**Figure 1:**
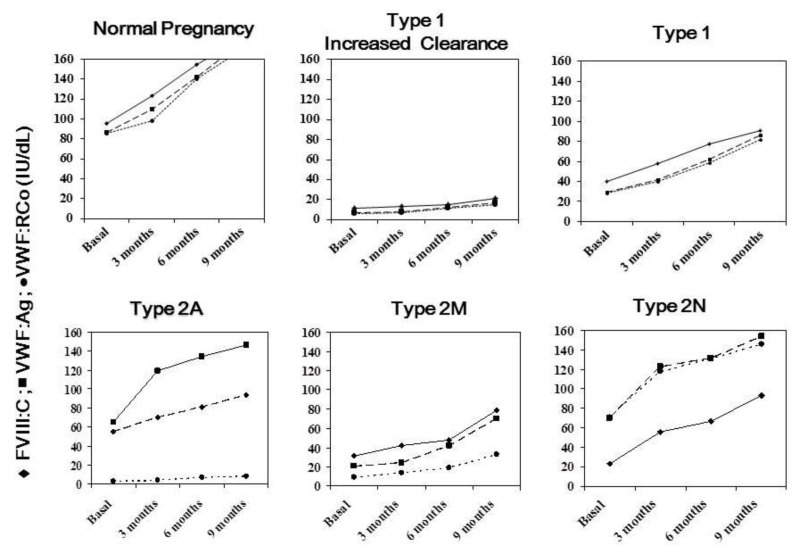
Modifications of FVIII and von Willebrand factor levels during normal pregnancy and in women with the more frequent types of von Willebrand disease From left to right: while during normal pregnancy an equivalent increase of all moieties occurs, several typical and different changes are observed in VWD. Patients with type 1 and increased VWF clearance do not show any significant improvement of their severely reduced baseline levels, while typical type 1 shows a progressive increase achieving normalization by the end of pregnancy and maintaining the normal 1:1 ratio between FVIII and VWF. In type 2A usually VWF:RCo remains markedly low compared to the increase of VWF:Ag and, by far more significant, of FVIII. In type 2M, the abnormal VWF:Ag/VWF:RCo remains unchanged because of small increase of VWF:RCo throughout. In homozygous type 2N, FVIII is normalized by the end of pregnancy, but its level remains significantly reduced compared to the largely increased VWF, which however maintain its reduced ability to bind FVIII.

Figure 1 summarizes the typical pattern of FVIII and VWF modifications occurring during normal pregnancy and in women with the more frequent types of VWD.

## Treatment of women with von Willebrand disease at delivery

Since pregnant women with VWD are at increased risk of postpartum hemorrhage if untreated,^12^,^14^–^16^ treatment options should be already planned at the beginning of pregnancy. Invasive management of delivery with ventouse, rotational forceps etc should be avoided for the risk of bleeding for the neonate potentially affected.^2^,^19^ Ideally, the results of a test-infusion with desmopressin should be available before pregnancy for every woman with VWD and basal level of FVIII and VWF <30 U/dL.^28^ However, choosing the treatment at parturition on the basis of basal levels alone, without knowledge of mutational background and/or the modifications of FVIII and VWF during pregnancy could be risky since several heterogeneous patterns are possible. In general, VWD patients should be monitored for VWF:RCo and FVIII:C at least once during the third trimester of pregnancy.^14^ The risk of bleeding is minimal when FVIII:C and VWF:RCo levels are around or higher than 50 U/dL.^19^,^29^,^30^

In type 1 VWD pregnant women with FVIII:C and/or VWF levels lower than 30 U/dL, the administration of desmopressin usually after umbilical clamping and for 3–4 days thereafter is required,^2^,^19^ especially when midline episiotomy is required. Urinary output and fluid restriction are advisable to avoid the risk of hyponatremia.^2^ The same approach, with less infusions can be applied to those with VWF >30 and <50 U/dL. A recent experience suggests the possibility to start treatment immediately before delivery, without evident side-effects for the mother and the newborn.^26^ The use of desmopressin during the first trimester of pregnancy to cover chorionic villus sampling or amniocentesis appears to be feasible and safe, without risk of miscarriage.^32^

In type 3 VWD women, VWF and FVIII do not increase during pregnancy and thus VWF/FVIII concentrates may be required during pregnancy to control intermittent vaginal bleeding and at delivery or for Cesarean section.^2^,^19^,^29^,^30^ The latter should be reserved only for the usual obstetrical indications. Daily doses of 50 IU/kg VWF are required to maintain FVIII:C level >50 U/dL for 3–4 days.^29^,^30^ Usual thrombo-prophylactic treatment with LMWH should be implemented in patients at high risk of venous thrombosis during replacement therapy for caesarean section.^2^ If performed, VWF:RCo and FVIII:C peak levels should be >50 U/dL.^29^,^30^

In patients with type 2A, 2B and 2M usually VWF:RCo does not attain normal levels and thus replacement therapy is similarly advised.^2^,^29^,^30^ However, in VWD 2B the increase of the abnormal VWF can cause or worsen thrombocytopenia^23^–^25^ and platelet concentrate transfusion could be also required at parturition.^33^

FVIII and VWF fall to baseline levels soon after delivery^16^,^17^,^19^ and thus oral antifibrinolytic agents (e.g., tranexamic acid 1 gr every 8 hours for 3–4 days) can be used during this period to prevent delayed postpartum bleeding due to heavy lochia.

## Figures and Tables

**Table 1:** Post-partum bleeding in type 1 von Willebrand disease women before Diagnosis (modified from Rodeghiero et al.^9^).

	Post-partum Bleeding Score*			
	0 No or trivial	1 Present, medical attention, iron therapy	2 Blood transfusion, dilatation, curettage, suturing	3 Hysterectomy
Type 1 VWD (n=37)	16 (43.2 %)	9 (24.3 %)	6 (16.2 %)	6 (16.2 %)
Controls (n=105)	102 (97.1 %)	1 (0.9 %)	2 (1.9 %)	0 -

* P < 0.001 for each grading between VWD patients and controls
